# Clinical Evaluation of a Novel and Mobile Autism Risk Assessment

**DOI:** 10.1007/s10803-016-2718-4

**Published:** 2016-02-12

**Authors:** Marlena Duda, Jena Daniels, Dennis P. Wall

**Affiliations:** Division of Systems Medicine, Department of Pediatrics, Stanford University, 1265 Welch Road, Stanford, CA 94305 USA; Department of Biomedical Data Science, Stanford University, 1265 Welch Road, Stanford, CA 94305 USA

**Keywords:** Autism screening, Autism detection, Machine learning, Clinical validation

## Abstract

The Mobile Autism Risk Assessment (MARA) is a new, electronically administered, 7-question autism spectrum disorder (ASD) screen to triage those at highest risk for ASD. Children 16 months–17 years (N = 222) were screened during their first visit in a developmental-behavioral pediatric clinic. MARA scores were compared to diagnosis from the clinical encounter. Participant median age was 5.8 years, 76.1 % were male, and most participants had an intelligence/developmental quotient score >85; 69 of the participants (31 %) received a clinical diagnosis of ASD. The sensitivity of the MARA in detecting ASD was 89.9 % [95 % CI = 82.7–97]; the specificity was 79.7 % [95 % CI = 73.4–86.1]. In a high-risk clinical setting, the MARA shows promise as a screen to distinguish ASD from other developmental/behavioral disorders.

## Introduction

Autism spectrum disorder (ASD) is characterized by significant impairments with social skills and communication, and atypical or repetitive behaviors (American Psychiatric Association and Task Force on DSM-IV 1994; Association 2013). The diagnosis is made clinically based on criteria described in the Diagnostic and Statistical Manual of Mental Disorders (DSM) (American Psychiatric Association and Task Force on DSM-IV 1994; Association 2013). Standardized assessment tools can be used to help operationalize the DSM criteria (Johnson and Myers 2007). ASD is reported to occur in up to 1 in 68 children (“Prevalence of autism spectrum disorder among children aged 8 years—autism and developmental disabilities monitoring network, 11 sites, United States, 2010, 2014“); thus, it represents a major public health issue. Although parents of children with ASD often report developmental concerns by the ages of 12–18 months, the average age of diagnosis in the United States is around 4 years (Zwaigenbaum et al. 2009). Screening tools can help to prioritize children at highest risk of ASD. Identifying those at highest risk may help facilitate more timely diagnostic assessments and access to evidence-based behavioral interventions, which have been shown to improve developmental and functional outcomes (Dawson et al. 2010; National Research Council (U.S.). Committee on Educational Interventions for Children with Autism 2001).

Screening tools can be used to detect normal development from abnormal development (Level 1) and, when developmental delays are suspected, to detect ASD from other developmental or behavioral conditions (Level 2) (Johnson and Myers 2007). With the relatively high prevalence of ASD, the use of Level 2 ASD screening tools to appropriately triage those who need more urgent diagnostic clarification is important. Several current Level 2 screening tools exist for detecting ASD (Norris and Lecavalier 2010) although most take considerable time to administer and require scoring to interpret (Johnson and Myers 2007). Some Level 2 screening tools, such as the Screening Tool for Autism in Two-Year-Olds (STAT) (Stone et al. 2008) and Autism Detection in Early Childhood (ADEC) (Nah et al. 2014), require the clinician to directly observe the child’s behavior while others, such as the Social Responsiveness Scale (SRS) (Constantino 2002), the Social Communication Questionnaire (SCQ) (Rutter et al. 2003), and Gilliam Autism Rating Scale-Second Edition (GARS-2) (Gilliam 2006) rely solely on parent report. While eliminating clinician observation time may be an advantage, the validity of parent report measures is dependent on how well the items assessed align with diagnostic criteria, and this can be influenced by the child’s age, developmental/intellectual level, and language abilities (Hampton and Strand 2015; Oosterling et al. 2010). The SRS (Constantino 2002) is a 65-item rating scales validated to distinguish ASD from other developmental conditions among children ages 4–18 years old (Constantino et al. 2000, 2003). Based on parent or teacher responses to the questions about symptoms of autism, a single score is generated, with higher score indicative of higher risk of the child having autism. The SCQ (Rutter et al. 2003) is another screening tool to discriminate between ASD cases and non-ASD cases in preschool and school-aged children. The SCQ consists of 40 yes/no questions that are based on the Autism Diagnostic Interview-Revised (ADI-R), which is a lengthy, parent interview that must be administered by a trained clinician (Lord et al. 1994). Questions remain regarding the optimal scoring threshold for the SCQ and whether some items should be adjusted based on the child’s language level (Eaves et al. 2006). Additionally, the performance of the SCQ when used to identify toddlers with ASD versus other developmental issues is greatly influenced by IQ, with sensitivity of 0.35 and specificity of 0.63 for toddlers with IQ > 90 (Oosterling et al. 2010). The GARS-2 (Gilliam 2006) is a 42-item parent questionnaire to screen for ASD among individuals 3–22 years of age. While psychometric properties have not yet been independently published for the second edition GARS (GARS-2), four of the five studies pertaining to the original GARS (Gilliam 1995) evaluated in a recent meta-analysis (Hampton and Strand 2015) found the GARS to have sensitivity and specificity levels below 70–80 %.

The current study sought to test a newly developed parent/caregiver completed Level 2 ASD screening tool, the Mobile Autism Risk Assessment (MARA). The MARA is brief and administered via an electronic platform with automatic scoring, thus decreasing barriers related to clinician training and time to score. Similar to the SCQ, the MARA stemmed from analysis of score sheets from the ADI-R but rather than clinical impression, machine learning techniques were employed to create this screener. Complete sets of answers to the ADI-R from the Autism Genetic Resource Exchange (AGRE) on 891 autism cases and 75 non-autism controls were used to build a series of classifiers from a set of different machine learning algorithms. The algorithm that performed the best was then independently validated using data from the Simons Foundation and the Boston Autism Consortium and it correctly identified a total of 1974 out of 1975 autistic cases (Wall et al. 2012). Although these results are promising, the MARA has not been studied prospectively, in a clinical setting, with a control sample of children with developmental disorders other than ASD. The primary objective of the current study is to test the sensitivity and specificity of the MARA in a clinical sample of children referred for developmental/behavioral concerns.

## Methods

### Setting and Participants

This study was conducted in the developmental-behavioral pediatrics clinic of a large academic medical center. Participants were children, ages 16 months–17 years, scheduled for their first diagnostic consultation visit to see a team of clinicians including a developmental- behavioral pediatrician and child psychologist, from November 2012 through December 2013. Referrals are generally made from pediatricians, early intervention agencies, school districts, and self-referrals. To obtain an appointment for a child, the guardian must complete paperwork stating the concerns and information about medical and developmental history and all those who complete this intake paperwork are scheduled for a clinic appointment; there is no screening process to deny visits. Children and adolescents are assigned to consultation clinic visits based on their age, rather than being assigned based on their referral concerns. The clinic population comes primarily from within the state of Massachusetts (86 %), with 9 % of those seen from other states within the United States and 5 % from other countries. Insurance type in the clinic is as follows: 60 % private, 37 % public, and 3 % self-pay. Whenever possible, caregivers were informed of the study via letter and a phone call prior to the clinic visit. Caregivers were directed to a secure website on which they could give electronic consent and complete the MARA. Initially recruitment was completed through letter and phone call only, but this method resulted in low enrollment numbers. Therefore, beginning 2 months after study initiation, a research assistant also approached caregivers in the waiting room prior to the beginning of the clinical visit and provided an iPad on which they could complete the MARA. Although financial compensation was not provided, as appreciation for completing the study all participating caregivers were entered into a raffle for the chance to receive an iPad. Non-English speaking caregivers were excluded given that the MARA questions are currently only available in English. This study received Institutional Review Board approval.

### Study and Clinical Measures

The MARA is a 7-item parent questionnaire about a child’s communication, social skills, and behaviors (Table 1). Each question has several accompanying sentences that the caregiver can consider in formulating answer choices. There are 4–5 answer choices available for each question, as well as the option of “not applicable”. Caregivers could complete the screener electronically on an iPad, computer, or any other device connected to the Internet. The set of answers is run through a machine learning model that uses an alternating decision tree algorithm to generate a total score (ranging from −10 to 7). This model was trained on 891 autism cases and 75 non-autism controls from the Autism Genetic Resource Exchange (AGRE) (Geschwind et al. 2001) repository and independently tested on archived samples from the Simons Simplex Collection and from the Boston Autism Consortium (Wall et al. 2012). The model used to train the classifier was an alternating decision tree. This approach finds features, in this case autism behaviors measured by ADI-R, that predict known class values, i.e. a person diagnosed with autism versus a person who was assessed and determined not to have autism. The power of the prediction was measured in terms of the number of correctly classified individuals during the training procedure, maximizing both precision and recall. The classifier was constructed using 10-fold cross validation, in which the data were divided evenly into 10-folds, and 90 % of the data (nine-folds) were dedicated to model training and the remaining 10 % (one-fold) was used to test the accuracy of the model. This process was repeated 10 times, until all combinations of training and testing folds had been exhausted. This resulted in a final classifier—a decision tree consisting of a collection of alternating prediction nodes and test nodes. The test nodes check whether a certain condition is true or not, for example whether a child scores high or low on a particular behavior. The predictor node predicts the likelihood of either an autism or non-autism classification. Classification is then achieved by summing the contributions from predictor nodes of all paths that an instance—in this case a set of data for a new child being screened—traverses.

**Table 1:** Mobile autism risk assessment (MARA) questions

1. How well does your child understand spoken language, based on speech alone? (Not including using clues from the surrounding environment)
2. Can your child have a back-and-forth conversation with you?
3. Does your child engage in imaginative or pretend play?
4. Does your child play pretend games when with a peer? Do they understand each other when playing?
5. Does your child maintain normal eye contact for his or her age in different situations and with a variety of different people?
6. Does your child play with his or her peers when in a group of at least two others?
7. When were your child’s behavioral abnormalities first obvious?

The behaviors measured by these 7 questions were identified from analysis of ADI-R score sheets using a decision tree learning model (Wall et al. 2012)

**Fig. 1:**
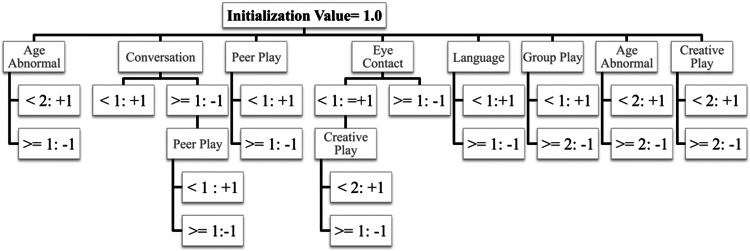
Representation of the MARA Algorithm. The alternating decision tree algorithm used for the MARA contains 7 total elements and 20 decision nodes. The outcome of either autism spectrum disorder or non-autism spectrum disorder is provided by the alternating decision tree by following all paths in the tree for which all decision nodes are true and summing the values. The numbers shown in the decision nodes are approximations of the fractional values contained in the algorithm

The decision tree algorithm used for the MARA contains 7 total elements (the questions in the MARA) and 20 decision nodes (the answers to the questions, some of which have been collapsed together) that either increase or decrease the total score depending on the answer provided by a parent or caretaker when taking the MARA. The outcome of either autism spectrum disorder or non-autism spectrum disorder is provided by the alternating decision tree by following all paths in the tree for which all decision nodes are true and summing the values. If the final score is negative, the instance is classified as autism spectrum disorder; if positive the instance is classified as non-autism spectrum disorder. Figure 1 depicts a representation of the decision tree classification system for the MARA. The magnitude of the score is a measure of confidence in the classification, such that values closer to 0 have lower confidence than values closer to the extremes of the distribution, a property of the alternating decision tree model (Wall et al. 2012). The MARA is written at a 7.9 grade reading level and takes about 5 min to complete.

Each subject participated in a multidisciplinary team clinic visit conducted by developmental-behavioral pediatricians and child psychologists, as per the routine for initial assessment in this clinic setting. The visit consisted of collection of complete medical and developmental history, physical examination, administration of developmental or cognitive measures, most commonly the Bayley Scales of Infant and Toddler Development, Third Edition (Bayley 2006), Differential Ability Scales, Second Edition (Elliott 2007) or the Wechsler Intelligence Scales for Children, Fourth Edition (Wechsler 2003), and behavior and/or adaptive functioning measures, such as the Vineland Adaptive Behavior Scales, Survey Interview Form (Sparrow et al. 1984). The Autism Diagnostic Observation Schedule (ADOS) was administered if an autism spectrum disorder was a diagnostic consideration in the clinical opinion of the team members or a significant concern of the caregivers. The decision of which tests to administer, and whether or not to administer the ADOS, was made either right before the clinical visit or during the course of the visit. After discussion of the results of the above-mentioned measures, clinical diagnoses were made by consensus agreement between the clinicians. During the study period, the updated Diagnostic and Statistical Manual of Mental Disorders-Fifth Edition (DSM-5) was published, with changes in diagnostic criteria for ASD. It is reasonable to assume that clinicians may have primarily conceptualized the DSM-IV-TR model for ASD in the first half of the study, and began to conceptualize the DSM-5 model for ASD once it was published in May 2013, which was mid-way through this study. Clinicians completed checklists assessing both the Diagnostic and Statistical Manual of Mental Disorders-Fourth Edition (DSM-IV-TR) and the new DSM-5 criteria for 62 % of the total sample because collection of these checklists was implemented as a Quality Improvement project that occurred concurrently with this study. In all cases, clinicians were blind to the results of the study screener to ensure that the clinical diagnoses and study screener results were independent. The ADI-R was not used for any of the clinical encounters, and is not routinely used in this clinical setting, thus eliminating possible confounding that could have occurred from using a screening tool which was developed from a diagnostic instrument administered to study participants (the ADI-R). Results of the clinical evaluation, including results of developmental or cognitive measures, verbal status, and clinical diagnoses made, were abstracted from the medical record.

### Data Analysis

Descriptive data about the sample were calculated using frequencies, *t* tests and Chi square values with accompanying *p* values. Chi square analyses were used to determine if the screener performed differently in those with ASD versus those without. Sensitivity and specificity were calculated to determine how well the screener performed, both in the whole sample and separately for different ages and developmental/cognitive abilities. Sensitivity was calculated as the proportion of all participants given a clinical ASD diagnosis who screened positive for ASD. Specificity was calculated as the proportion of all participants not given a clinical ASD diagnosis who screened negative for ASD. The positive predictive value was calculated as the likelihood that a person with a MARA result indicative of ASD actually received a clinical ASD diagnosis. The negative predictive value was calculated as the likelihood that a person with a MARA result negative for ASD did not actually receive a clinical ASD diagnosis.

## Results

### Descriptive Results

**Fig. 2:**
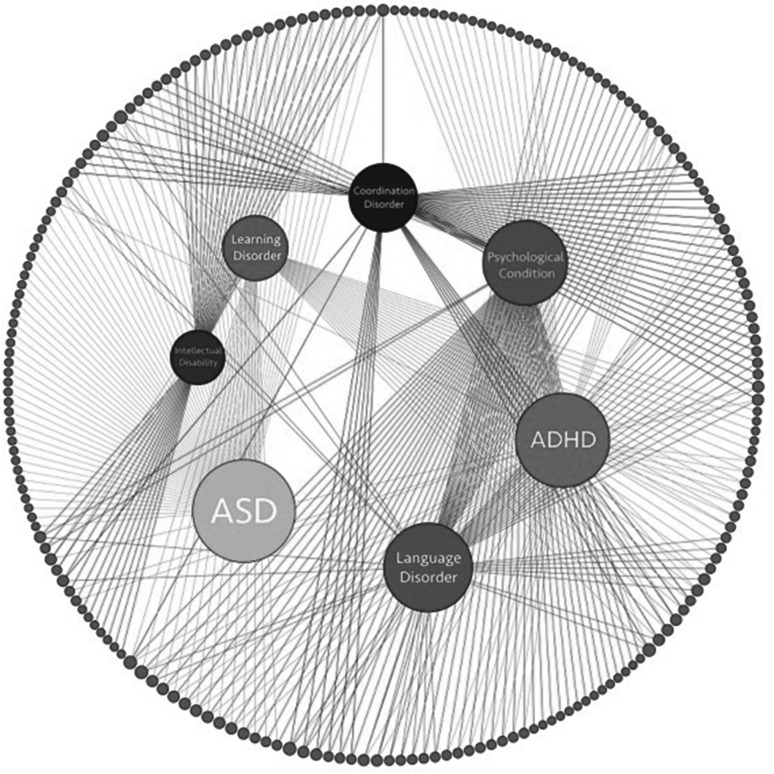
Diagnosis overlap network across our clinical sample. Network visualization of diagnoses across our clinical sample (n = 222). *Outer grey* nodes represent individual subjects in our sample and *inner colored* nodes represent the seven major diagnostic categories observed. *Edges* connecting *inner* nodes to *outer* nodes indicate that subject received that diagnosis. *Outer* nodes with multiple connections indicate subjects with multiple comorbid diagnoses. 10 subjects in our sample did not receive any diagnoses in these seven major categories, but may have received other less common diagnoses (Color figure online)

A total of 222 participants completed the MARA and then participated in the clinical visit, representing 46 % of those invited to participate in the study. This relatively low enrollment rate reflects the low yield of the initial recruitment strategy (i.e. mail contact) employed in the initial 2 months of the study. Given the resulting low enrollment rate, we changed to in-person recruitment in the clinic waiting room (rather than relying on subjects participating remotely prior to their clinical encounter) with a much improved enrollment rate. The majority of subjects (N = 213; 95.95 %) were enrolled via in-person recruitment; 6 subjects (2.7 %) were enrolled via letter and phone call recruitment efforts, and only 3 subjects (1.3 %) were enrolled via letter recruitment efforts alone. There was no significant difference in median age, gender, or receipt of a clinical ASD diagnosis between those who participated and those who did not. The median age of participants was 5.8 years, 76.1 % were male, and 66.7 % of those with non-verbal IQ data available in the medical record (n = 117) had a non-verbal cognitive score >85. For the remaining subjects (N = 105), standardized scores were not reported or full developmental/cognitive testing was not completed, most commonly because it had been completed elsewhere within the past year, there was a need to focus on other assessments, or insurance did not cover full testing. For subjects who had data about verbal status abstracted from the medical record (N = 215), 198 subjects were verbal and 17 subjects were non-verbal. For 31 subjects caregivers reported an existing ASD diagnosis prior to the multidisciplinary team consultation. Of all participants, 69 (31 %) were given a clinical diagnosis of an autism spectrum disorder and the remaining 153 were given other clinical diagnoses (such as Attention Deficit Hyperactivity Disorder and Speech Delay/Language Disorder). Figure 2 shows the distribution and overlap of the seven most frequently identified diagnostic categories in our sample. Participants who were given a clinical ASD diagnosis were more likely to be male, younger age, and have intellectual or developmental delays compared to those given other clinical diagnoses (Table 2). The Autism Diagnostic Observation Schedule (ADOS) was administered in 67 of the 69 cases in which a clinical ASD diagnosis was made and, in all of these cases, supported the clinical ASD diagnosis. The ADOS was not administered to 2 children given a clinical diagnosis of ASD based on clinician judgment that the child would not tolerate the testing or there was not sufficient time to complete it. For 50 subjects, clinicians recorded which DSM-IV-TR sub-group diagnosis was given as follows: 86 % Autistic Disorder, 12 % Pervasive Developmental Disorder, not otherwise specified, 2 % Asperger’s Disorder. For the remaining 19 subjects, the clinical diagnosis was recorded as “autism spectrum disorder” with no DSM-IV-TR sub-type identified. Of the 69 participants given a clinical ASD diagnosis, 50 had information available about specific criteria met on both DSM-IV-TR and DSM-5 criteria and 92 % met criteria for ASD under both sets of criteria. During the course of this study the new DSM-5 criteria were released.

**Table 2:** Descriptive information about total sample and those with versus without an autism spectrum disorder diagnosis

	Total sample (N = 222)	Clinical ASD diagnosis (N = 69)	No ASD diagnosis (N = 153)	Difference between ASD versus no-ASD *p* value*
Gender				
Male	169 (76.1 %)	60 (87.0 %)	109 (71.2 %)	0.018
Age in years				
Median (IQR)	5.8 (4.6)	3.9 (3.3)	6.6 (3.9)	<0.0001^1^
Other clinical diagnoses				
ADHD, any sub-type	58 (26.13 %)	1 (1.4 %)	57 (37.2 %)	<0.0001
Speech delay/language disorder	59 (26.58 %)	4 (5.8 %)	55 (36.0 %)	<0.0001
Developmental coordination disorder	43 (19.36 %)	7 (10.1 %)	36 (23.5 %)	0.0314
Learning disorder	42 (18.92 %)	2 (2.9 %)	40 (26.1 %)	<0.0001
Mood disorder	2 (0.90 %)	0	2 (1.3 %)	0.8519
Depression	5 (2.25 %)	0	5 (3.3 %)	0.3029
Anxiety disorder	33 (14.86 %)	3 (4.3 %)	30 (19.6 %)	0.0059
Hearing or vision impairment	3 (1.35 %)	1 (1.4 %)	2 (1.3 %)	0.9324
Genetic condition	6 (2.70 %)	2 (2.9 %)	4 (2.6 %)	0.9038
Global developmental delay/intellectual disability	24 (10.81 %)	20 (29.0 %)	15 (9.8 %)	0.0006
Other medical condition	78 (35.14 %)	13 (18.8 %)	65 (42.5 %)	0.0011
Developmental/IQ score^a^ Median (IQR)				
Full Scale IQ	97.0 (22.0)	96.0 (30)	97.5 (20.8)	0.6212^1^
Non-verbal IQ	91.0 (22.0)	87.5 (20.8)	95.0 (19.0)	0.0914^1^
Verbal IQ	94.0 (24.0)	87.0 (36.5)	96.0 (21.3)	0.0996^1^

* Chi square was test statistic used unless otherwise indicated

^1^Wilcoxon rank-sum test used to assess for differences in groups

^a^Developmental/IQ score had some missing data; N = 105 subjects had Full Scale IQ data, N = 117 subjects had non-verbal IQ data, N = 129 subjects had verbal IQ data available

*ASD* autism spectrum disorder, *ADHD* attention deficit hyperactivity disorder, *IQR* interquartile range, *IQ* intelligence quotient

### Performance of the MARA

MARA scores were dichotomized to be high or low risk for ASD based on previously established cut-offs (Wall et al. 2012). The MARA scores in this study ranged from −9.28 to 6.43, with negative scores indicating high risk and positive scores suggesting low risk for ASD. Subjects who received a clinical ASD diagnosis were more likely than those without a clinical ASD diagnosis to receive a MARA score that was indicative of ASD (*x*^2^ = 91.77, *p* < 0.0001). Overall, the sensitivity of the MARA in detecting ASD was 89.86 %, 95 % CI [82.7, 97.0] and the specificity was 79.74 %, 95 % CI [73.4, 86.1]. Table 3 demonstrates the performance of the MARA across different ages and different developmental/cognitive abilities, showing a higher specificity for those with cognitive/developmental scores that were in the low average range or higher (≥85). Among subjects who were non-verbal (N = 17) the MARA had a high sensitivity (1.0) for detecting ASD, but misclassified 3 out of the 4 non-verbal subjects as having ASD (specificity = 0.25). For the total sample, the positive predictive value was 0.67 and the negative predictive value was 0.95. Although the subjects in the study were those being seen for a multidisciplinary diagnostic consultation there were 31 subjects for whom caregivers reported an existing ASD diagnosis prior to receiving the results of the diagnostic consultation. Therefore, in a post hoc analysis we excluded these subjects and among the 191 remaining subjects the MARA performed with sensitivity of 89.58 % and specificity of 80.42 %. As an additional post hoc analysis, we evaluated whether the performance of the screener varied based on whether the caregiver completed it at home or in the clinic and we did not see any difference in screening performance based on location of completion (*x*^2^ = 5.63, *p* = 0.13).

**Table 3:** Performance of the MARA across different ages and cognitive/developmental levels

	# Subjects	# Subjects with clinical ASD diagnosis^a^	Sensitivity [95 % CI] (%)	Specificity [95 % CI] (%)
Total sample	222	69	89.9 [82.7–97]	79.7 [73.4–86.1]
Age <3 years	38	25	96 [88.3–100]	61.5 [35.1–88]
Age 3–6 years	103	33	84.8 [72.6–97.1]	75.7 [65.7–85.8]
Age >6 years	81	11	90.9 [73.9–100]	87.1 [79.3–95]
Cognitive/development score^a^ <70	15	7	100 [100–100]	62.5 [29–96]
Cognitive/development score^a^ 70–84	24	14	78.6 [57.1–100]	50 [19–81]
Cognitive/development score^a^ 85–100	46	19	100 [100–100]	70.4 [53.1–87.6]
Cognitive/development score^a^ >100	32	12	75 [50.5–99.5]	80 [62.5–97.5]

^a^Cognitive/development score is based on non-verbal IQ for whom N = 117 subjects had available data

*MARA* Mobile Autism Risk Assessment

**Fig. 3:**
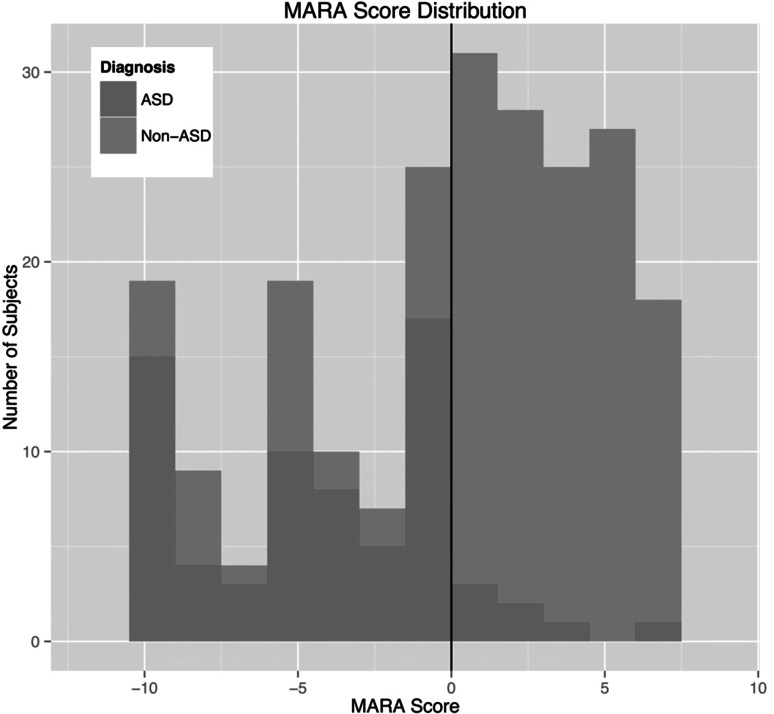
MARA score distribution. This histogram shows the distribution of MARA scores for those with ASD compared to those without ASD. The *line* at 0 represents the classification cutoff for the MARA algorithm—individuals with a MARA score <0 are classified as ASD and individuals with a MARA score >0 are classified as non-ASD using this screener

There were 31 subjects who screened positive for ASD but were not given a clinical diagnosis of ASD. The most common clinical diagnoses for these subjects were Language Delay/Disorder (48.4 %), Motor Delay/Coordination Disorder (38.7 %), and Global Developmental Delay/Intellectual Disability (25.8 %). There was no significant difference in IQ between the correctly classified and misclassified groups (*p* = 0.8304). Misclassified subjects were more likely to be diagnosed with a language delay/disorder compared to correctly classified subjects (44.7 vs. 22.9 %; *p* = 0.01). There were 7 subjects who screened negative for ASD but were given a clinical diagnosis of ASD. These subjects all had an IQ ≥ 84 and most had MARA scores that were close to the cut-off for ASD; specifically, 5 had a fairly low score (<3) on the MARA. Figure 3 shows a histogram of the MARA scores for ASD and non-ASD diagnoses.

## Discussion

The current study demonstrates that the MARA autism screener performs well (sensitivity = 89.9 % and specificity = 79.7 %) in detecting children likely to receive a clinical diagnosis of ASD among those referred to a tertiary care center for developmental or behavioral concerns. When tested among 222 subjects, with a range of different ages (median age = 5.8 years) and abilities, the MARA performed best for subjects with an IQ of 85 or higher.

In its current form, the MARA is considered a Level 2 screening tool, meaning that it is meant to differentiate between children at risk for ASD and other developmental disorders. When compared to several other ASD specific Level 2 caregiver questionnaire screeners in use (Johnson and Myers 2007), the MARA has comparable or superior ability to detect ASD among children with developmental or behavioral concerns. Of currently available Level 2 ASD rating scales, the Social Communication Questionnaire (SCQ) (Rutter et al. 2003) has been most extensively studied (Norris and Lecavalier 2010). The SCQ is reported to be able to discriminate between ASD cases and non-ASD cases with 88 % sensitivity and 72 % specificity (Chandler et al. 2007) although it is reported to have lower sensitivity and specificity for detecting ASD in pre-school aged children (Eaves et al. 2006). The Social Responsiveness Scale (SRS) (Constantino 2002) is a commonly used parent report measure to assess likelihood of ASD and it is reported to have sensitivity ranging from 75 to 95 % and specificity ranging from 8 to 96 % (Hampton and Strand 2015). The Gilliam Autism Rating Scale (GARS) (Gilliam 1995) is another commonly used parent measure of ASD, despite sensitivity reported to be 37–79 % and specificity reported to be only 54–68 % (Hampton and Strand 2015). The great range in reported sensitivities and specificities for different screening tools is likely in part attributable to different sample compositions, as screeners may perform differently for different ages, and for those with different developmental/cognitive abilities (Hampton and Strand 2015; Oosterling et al. 2010). Therefore, further studies assessing the MARA will include larger sample sizes to allow for more informative psychometric information about how the MARA performs among children and adolescents of differing ages and with differing developmental presentations. If it is found to perform well in a larger validation study, the MARA may be particularly useful in secondary screening efforts since it is administered via an electronic platform with automatic scoring that decreases clinician training needed to implement, and increases potential ability for dissemination as it can easily be completed remotely. However, the need for electronic scoring could potentially be seen as a disadvantage so future studies will need to evaluate the feasibility of implementation of the MARA across diverse clinical settings.

Although the MARA stemmed from analysis of score sheets from the Autism Diagnostic Interview-Revised (Lord et al. 1994) it is not meant to replace a diagnostic encounter. Instead, if our findings are replicated in larger clinical samples, the MARA could serve as a triage tool to help identify children with developmental and/or behavioral concerns that are highest risk for meeting criteria for ASD in order to expedite their diagnostic evaluation and receipt of behavioral interventions. Our results show that those who were misclassified by the MARA as high likelihood for ASD (but not given a clinical ASD diagnosis) were most often diagnosed with language delays or disorders, which is not surprising given the clinical overlap that often exists between children with ASD versus language delays or disorders. Of the 7 subjects who were “missed” by the screener, most were fairly close to the cut-off for ASD (5 of the subjects had a MARA score <3) and would be flagged as challenging cases requiring more extensive assessment in clinical implementation of this screening measure.

Although the preliminary findings of the psychometric properties of the MARA in a clinical setting are encouraging, these findings are not as robust as those initially reported in the pilot study run on archival samples (Wall et al. 2012). The pilot focused on construction of a classifier optimized for performance with classifying autism spectrum disorder from controls. This study had several limitations, most importantly including the high prevalence of classic, DSM-IV Autistic disorder in the archival samples used for validation and the lack of testing on children with other forms of autism spectrum disorder (e.g. PDD-NOS) or with developmental delays other than autism spectrum disorder. Therefore the drop in accuracy, in particular the decline in specificity exhibited here, is expected given the large proportion of children in our study with developmental delays other than autism spectrum disorder. Additionally, in the prior pilot study (Wall et al. 2012), the data were obtained through results of clinical ADI-R interviews administered by trained interviewers whereas, in the current study, the data were obtained through parental responses, and thus a discrepancy in responses in the different studies may be expected.

The findings of our study must be considered in the context of some potential limitations. Our study was conducted at only one large academic medical center, thus potentially limiting generalizability of the results. However, evaluating the MARA in a high risk setting that specializes in evaluating children with a range of developmental and behavioral concerns enabled us to test the specificity of the MARA in detecting ASD versus other developmental conditions. Future studies should evaluate the MARA across diverse clinical settings. This study was conducted at the time of transition between DSM-IV-TR and DSM-5 ASD criteria. Although specific information collected for a subset of patients did not reveal significant variation in diagnostic outcomes based on use of DSM-IV-TR versus DSM-5 criteria, it is possible that the change in criteria will result in some changes in diagnostic practice. Thus, future studies that occur once DSM-5 criteria are fully operationalized in clinical practice will be important. Additionally, information was not available on how the changing DSM criteria may have influenced comorbidities in the sample. For those who were not diagnosed with ASD, several other clinical diagnoses were made, most commonly ADHD, speech delay/language disorder, learning disorders, and other medical conditions. The heterogeneity in clinical diagnoses given has important implications for interpretation of specificity (i.e., differentiating ADHD or speech delay/language disorder from ASD is more challenging than differentiating learning disorder or other medical conditions from ASD) thus future studies can further assess the specificity of the MARA in a more diagnostically homogeneous clinical population.

Nonetheless, in this initial study the performance of the MARA performed sufficiently to warrant further evaluation. In assessing the performance of the MARA in different groups based on cognitive/developmental level and age, small sample sizes in some groups warrant cautious interpretation. In particular, there were relatively few participants in this study with developmental/IQ level <70 (N = 15 participants) and there were also relatively few young participants (N = 38 participants less than 3 years old). Although the sensitivity was high in these groups, specificity was relatively low and larger sample sizes must be studied as a next step. Additionally, the ASD and non-ASD groups differ significantly on both age and percentage with developmental/intellectual delays and these differences limit the interpretation of the sensitivity and the specificity. The relatively large percentage of subjects with missing development/IQ scores reported also limits the interpretation of these findings. Future studies can further investigate if the MARA performs more robustly for certain ages and/or cognitive/developmental levels. We were not able to obtain specific information about the socioeconomic status of participants in this study, although information about the insurance status are known for the clinic in general and reported in the methods section. Despite these limitations, our findings support further evaluation of the MARA for potential widespread dissemination as a secondary screener to assess developmental concerns if it continues to perform well in larger, diverse clinical samples.

When tested in a clinical sample of 222 subjects with median age of 5.8 years and most with intact cognitive abilities (cognitive/developmental score >85), this new ASD screening tool (the MARA) demonstrated good ability to distinguish ASD versus other developmental and behavioral concerns. The electronic platform, brief administration time and automatic scoring increase its potential for widespread use as a secondary ASD screening tool if further studies support these findings.
